# Improvement of Bone Mineral Density in Patients with Type 1 Gaucher Disease Treated with Velaglucerase Alfa: Results from Clinical Studies

**DOI:** 10.3390/jcm15072537

**Published:** 2026-03-26

**Authors:** Ari Zimran, Jaco Botha, Richard Eastell, Can Ficicioglu, Richard D. Finkelman, Dafna Frydman, Pilar Giraldo, Ozlem Goker-Alpan, Priya S. Kishnani, Heather Lau, Noa Ruhrman-Shahar, Derralynn A. Hughes

**Affiliations:** 1 Gaucher Unit, Shaare Zedek Medical Center, Jerusalem 9103102, Israel; azimran@gmail.com (A.Z.); dafnaf@szmc.org.il (D.F.); 2 Faculty of Medicine, Hebrew University, Jerusalem 9112102, Israel; 3 Takeda Pharmaceuticals International AG, 8152 Opfikon, Switzerland; jaco.botha@takeda.com; 4 Academic Unit of Bone Metabolism, University of Sheffield, Sheffield S5 7AU, UK; r.eastell@sheffield.ac.uk; 5 Division of Human Genetics and Metabolism, The Children’s Hospital of Philadelphia, Philadelphia, PA 19104, USA; ficicioglu@email.chop.edu; 6 Perelman School of Medicine, University of Pennsylvania, Philadelphia, PA 19104, USA; 7 Takeda Development Center Americas, Inc., Cambridge, MA 02142, USA; richard.finkelman@takeda.com; 8 Translational Research Unit, IIS Aragon, 50009 Zaragoza, Spain; giraldocastellano@gmail.com; 9 CIBER de Enfermedades Raras, IIS Aragon, 50009 Zaragoza, Spain; 10 Lysosomal and Rare Disorders Research and Treatment Center, Fairfax, VA 22030, USA; 11 Division of Medical Genetics, Department of Pediatrics, Duke University Medical Center, Durham, NC 27710, USA; 12 Department of Internal Medicine, University of Yale, New Haven, CT 06520, USA; 13 The Raphael Recanati Genetics Institute, Rabin Medical Center—Beilinson Hospital, Petah Tikva 49100, Israel; 14 Lysosomal Storage Disorders Unit, Royal Free London NHS Foundation Trust, University College London, London NW3 2QG, UK

**Keywords:** velaglucerase alfa, Gaucher disease, bone mineral density, bone marrow burden, enzyme replacement therapy, glucocerebrosidase

## Abstract

**Background:** Despite the availability of effective therapies for Gaucher disease (GD), management of skeletal disease manifestations remains challenging. **Methods:** This phase 4 SHP-GCB-402 study evaluated the effect of velaglucerase alfa on lumbar spine (LS) bone outcomes in patients with type 1 GD. **Results:** Twenty-one patients with documented bone pathology received ≥1 dose of velaglucerase alfa 60 U/kg; 16 completed this study. The primary endpoint—change from baseline to 24 months in an LS bone mineral density (BMD) Z-score measured by dual-energy X-ray absorptiometry—showed a numerical improvement from baseline (mean [SD] −1.93 [0.88]) to 24 months (−1.76 [1.00]), although statistical significance was not reached (*p* = 0.1077). These changes are not consistent with previous velaglucerase alfa studies. To contextualize these findings, a pooled analysis of 24-month data from previous velaglucerase alfa trials was conducted. In this cohort (*n* = 40), a statistically significant mean (SD) increase in the LS BMD Z-score of 0.55 (0.58; *p* < 0.0001) was observed, supporting the therapeutic potential of velaglucerase alfa in improving skeletal outcomes. Additionally, SHP-GCB-402 demonstrated a significant reduction in the bone marrow burden (BMB) score (mean change from baseline: −3.0 [2.27]; *p* = 0.0005), indicating a positive effect on bone marrow infiltration. All patients experienced ≥1 treatment-emergent adverse event, mostly of mild/moderate severity. **Conclusions:** The observed numerical improvements in BMD and significant improvements in BMB in SHP-GCB-402 along with pooled BMD data suggest that velaglucerase alfa may confer skeletal benefits while maintaining a consistent safety profile.

## 1. Introduction

Gaucher disease (GD) is a rare autosomal recessive lysosomal disorder caused by pathogenic variants in the glucocerebrosidase gene (*GBA1*), resulting in deficient β-glucocerebrosidase enzyme activity. This leads to the accumulation of glucocerebroside in the lysosomes of cells of the monocyte/macrophage system, primarily in the liver, spleen, and bone marrow [1], inducing a cascade of pathological responses, including macrophage activation and inflammation, inhibition of osteoblast activity, and increased osteoclast activation and bone resorption [2]. Impaired function of mesenchymal stem cells and development defects in osteoblasts have also been observed in patients with GD [3,4].

GD has a variable phenotypic presentation and is broadly classified into three clinical subtypes [5], of which type 1 (GD1) is the most common in populations from Europe and North America [6]. GD1 is characterized by multisystemic clinical manifestations, including splenomegaly, hepatomegaly, thrombocytopenia, anemia, and bone disease [2,7]. Skeletal manifestations in patients with GD1 are more common in adults who were not treated with GD-specific therapies as children, suggesting onset during bone formation in childhood. If left untreated, they may become painful and debilitating and significantly affect patients’ quality of life [7]. Bone involvement includes bone crises, osteosclerosis, cortical thinning, lytic bone lesions, skeletal remodeling abnormalities, decreased bone mineral density (BMD), Erlenmeyer flask deformity, avascular osteonecrosis, and pathological fractures, leading to bone pain and disability [2,7]. In an analysis of data from the International Collaborative Gaucher Group registry, 94% of patients were found to have radiological evidence of bone disease [8], with the most common pre-treatment bone features found to be bone pain (63%), Erlenmeyer flask deformity (46%), osteopenia (42%), marrow infiltration (40%) and bone crises (33%) [8].

Despite the availability of effective treatment for GD since 1991 [7], the management of skeletal manifestations of the disease remains challenging. In particular, osteonecrosis remains one of the most problematic morbidities of bone involvement for adults with GD, albeit with a significant reduction in prevalence since the availability of enzyme replacement therapy. GD-related bone disease affects both the bone marrow, assessed using the bone marrow burden (BMB) score from MRI imaging [7], and BMD, assessed by dual-energy X-ray absorptiometry (DXA), the latter widely considered a reliable measure of bone strength [9,10]. The effects of velaglucerase alfa on BMD in patients with GD1 have been evaluated in three clinical studies; the first-in-human TKT025 phase 1/2 study and the TKT025EXT extension study [11], and the phase 3 TKT032 [12] and HGT-GCB-039 [13] studies, and the subsequent extension study (HGT-GCB-044) [14]. In TKT025EXT, statistically significant increases in BMD Z-scores were observed in the lumbar spine (LS) after 24 months of 60 U/kg velaglucerase alfa treatment every other week (EOW) and in the femoral neck after 33 months in 10 adults with GD1 [15]. In HGT-GCB-044, statistically significant mean increases in LS BMD Z-score were observed up to 24 months after treatment initiation with velaglucerase alfa therapy at a dose of 60 U/kg [12,13,14]; however, no statistically significant change in BMD was seen in the femoral neck for these patients, consistent with previous studies that suggest longer treatment durations are required to achieve detectable improvements at this location [16,17]. Despite these promising early bone data from phase 1/2 and extension studies, phase 3 studies with velaglucerase alfa included bone parameters as exploratory rather than secondary endpoints; therefore, there was a need for a further study to evaluate the effect of velaglucerase alfa on GD-related bone disease.

This phase 4 SHP-GCB-402 study was conducted to assess the effect of velaglucerase alfa on spinal BMD in patients with low BMD at baseline. However, notable differences in demographic and disease characteristics emerged between patients in SHP-GCB-402 and those enrolled in earlier velaglucerase alfa trials. Patients in SHP-GCB-402 were, on average, older and were enrolled under broader eligibility criteria that did not require a minimum threshold for non-skeletal GD manifestations. As a result, the study population included patients with comparatively milder hematologic and visceral involvement than in previous clinical trials. To account for these differences and better contextualize the bone-related findings, a pooled analysis of 24-month data from previous velaglucerase alfa clinical studies was undertaken. Based on previous studies that have identified age, sex, genotype, and time from diagnosis to treatment initiation as variables affecting BMD [18,19], further subgroup analyses of data from SHP-GCB-402 were performed to examine their influence on BMD outcomes and to offer a clearer context for interpreting the clinical data.

## 2. Methods

### 2.1. Study Design

SHP-GCB-402 was a 24-month, multinational, open-label, single-arm study (ClinicalTrials.gov: NCT02574286, EudraCT: 2015-001578-17) conducted in 8 medical centers in Israel, Spain, UK, and the United States between 7 June 2016 and 30 November 2020 (Table 1). This study comprised a 28-day screening/baseline period, a 24-month treatment period (51 infusions), followed by a 30-day safety follow-up period. It was anticipated that 24 months of treatment with velaglucerase alfa would be sufficient to observe statistically significant changes in LS BMD Z-scores, based on previous experience in Study TKT025EXT [11] and Study HGT-GCB-044 [14,20].

The protocol was approved by the institutional review board or independent ethics committee of each participating site. This study was conducted in accordance with the International Conference on Harmonization Good Clinical Practice guidelines, and all patients provided written informed consent before study enrollment.

### 2.2. Patients

Patients aged ≥ 18 and ≤70 years with a documented diagnosis of GD1 determined by deficient glucocerebrosidase activity in either leukocytes (whole blood only) or cultured skin fibroblasts were eligible for inclusion in this study. Patients were required to have an LS BMD Z-score < −1 or a BMD T-score of <−1 as measured by DXA during screening. Previous enzyme replacement therapy or substrate reduction therapy for GD in the 12 months before enrollment was not permitted. No inclusion criteria relating to the presence or severity of specific GD1-related manifestations were specified. Additionally, there were no inclusion criteria relating to a minimum threshold for non-skeletal GD-related symptoms. Patients were excluded from this study if they had neurologic symptoms consistent with type 3 GD or any significant comorbidity or joint-associated bone damage that might impact study participation or compromise study assessment. Patients who received any osteoporosis-specific treatment (e.g., bisphosphonates) or erythropoietin (or erythropoietin-like substances) in the 12 months before enrollment or who had undergone splenectomy were also excluded. Patients with vitamin D insufficiency at screening (i.e., concentration of 25-hydroxy vitamin D [25(OH)D] > 10 ng/mL [25 nmol/L] and <30 ng/mL [75 nmol/L]) could be treated with vitamin D (4000 IU per day) for 1 month and then re-screened for study entry. Patients with vitamin D deficiency (25[OH]D < 10 ng/mL [25 nmol/L]), consistent with the development of osteomalacia, were excluded.

### 2.3. Study Treatment

All patients received 60 U/kg velaglucerase alfa EOW administered as a 60-min intravenous infusion. Infusions were scheduled to occur on approximately the same day of the week but could occur every 14 days ± 7 days. Body weight (BW) was measured every 12 weeks throughout the study, a ±5% change in BW from the last measurement required dose recalculation, with any dosing adjustment applied to the next dose. After the first 3 doses, patients who had not experienced a treatment-related serious adverse event or an infusion-related adverse event of more than mild severity could receive subsequent infusions at home administered by qualified and trained medical personnel.

### 2.4. Study Endpoints and Assessments

The primary endpoint was the change in LS BMD Z-score from baseline to 24 months. BMD was measured in the L1-4 or L2-4 region using DXA (Horizon® DXA System Hologic Inc., Marlborough, MA, USA or Lunar iDXA or Prodigy, GE Lunar Corp. GE Healthcare Inc., Marlborough, MA, USA, depending on the site) and converted to Z-scores appropriate for patients’ age, sex, and race. Device calibration procedures and image collection, preparation, and transfer instructions were provided to clinical sites. Scans were analyzed centrally by VirtualScopics (BioTelemetry, Inc.), Rochester, NY, USA, or Geneva, Switzerland, with repeat scans undertaken using the same equipment (the 3 assessments for each patient were conducted using the same machine and software). Assessments on femoral neck BMD were not performed based on previous data suggesting treatment durations beyond the period of study required for a detectable response.

Secondary bone-related endpoints were change in LS BMD g/cm^2^, i.e., not normalized to age, sex and race, change in total BMB score of the LS and femur measured using MRI with results scored as 0–16 [21] and World Health Organization (WHO) BMD classification (normal bone density, osteopenia, osteoporosis) based on LS BMD T-scores [22,23]. Secondary efficacy endpoints included change in hemoglobin concentrations, platelet counts, and liver and spleen volumes as measured by abdominal MRI. Safety outcomes were also assessed as a secondary endpoint, and treatment-emergent adverse events (TEAEs) were monitored throughout the study. Exploratory endpoints included change in lyso-Gb1, currently considered the most specific and most sensitive biomarker for GD [24,25].

### 2.5. Statistical Analyses

The intent-to-treat population for efficacy analyses and the safety population were both defined as patients who received ≥ 1 study drug infusion (full or partial). Primary endpoint analysis used all observed cases (completer population at month 24); no missing data imputation was performed. Post hoc subgroup analyses were conducted on the primary outcome measure of mean change from baseline in LS BMD Z-score to 24 months for subgroups including sex, time from diagnosis to treatment initiation, and splenomegaly status. All data were summarized and presented using descriptive statistics with continuous variables summarized as mean (standard deviation [SD]) and median (range) and categorical variables as frequencies and percentages. Summary statistics were reported for change from baseline for each parameter. Two-sided 95% confidence intervals (CIs) in the mean changes from baseline are presented for each endpoint. Within-patient changes from baseline were examined using 1 sample *t* test. Statistical significance was defined at the 0.05 level. All statistical analyses were performed using SAS® software, version 9.3 or higher (SAS Institute, Cary, NC, USA).

### 2.6. Sample-Size Calculation

This sample-size calculation was based on the assumptions of a 2-sided *t* test with an alpha of 0.05, and an SD of the change in LS BMD Z-scores at 24 months of 0.6, as observed in Study HGT-GCB-044. It was calculated that a total of 13 evaluable patients would provide 90% power to detect a significant change in LS BMD Z-score from baseline after 24 months of treatment. Assuming 30% early discontinuation up to 1 year, at least 19 patients were planned for enrollment.

### 2.7. Pooled Analyses

In order to further contextualize available bone data with velaglucerase alfa treatment among different study populations, a pooled analysis of 24-month BMD data from previous clinical trials was conducted. The pooled analysis included data from 3 velaglucerase alfa clinical studies that enrolled patients with confirmed GD1 who were naïve to GD-specific treatment and included the LS BMD Z-score as an exploratory endpoint: the phase 1/2 TKT025 study and its extension study [11], and the phase 3 TKT032 [12] and HGT-GCB-039 studies [13], together with the subsequent extension study (HGT-GCB-044) (Table 1) [14]. From each of the 3 eligible studies, treatment naïve patients aged ≥ 18 years at the start of the first study period who received ≥1 dose of velaglucerase alfa and had LS BMD Z-scores up to 24 months were included. The studies and their extension studies were conducted between April 2004 and December 2012. Methods for TKT025, TKT025EXT, TKT032, HGT-GCB-039, and HGT-GCB-044 have been reported in full previously [11,12,13,14]. In TKT025, the first 3 enrolled patients received escalating doses of velaglucerase alfa, from 15–60 U/kg EOW. Patients continuing into TKT025EXT received 60 U/kg EOW [11]. In TKT032, patients were randomized to receive velaglucerase alfa 45 or 60 U/kg for 51 weeks before continuing treatment with velaglucerase alfa 60 U/kg in HGT-GCB-044 up to a total of 24 months [12,14]. Patients received 60 U/kg velaglucerase alfa EOW for 39 weeks in the HGT-GCB-039 study, before continuing treatment in HGT-GCB-044 up to a total of 24 months [13,14].

## 3. Results

### 3.1. SHP-GCB-402 Study

#### 3.1.1. Patient Baseline Characteristics

**Figure 1:**
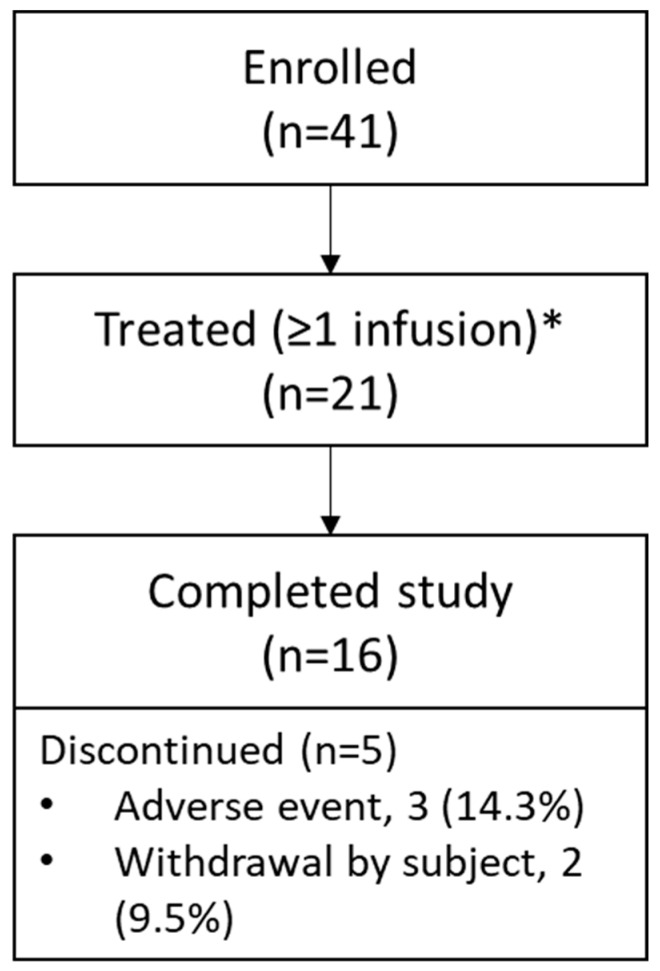
Patient disposition, SHP-GCB-402 study. * Intent-to-treat and safety populations.

Twenty-one patients were enrolled and received ≥1 dose of velaglucerase alfa 60 U/kg and 16 completed the study. Of the five patients who discontinued, two (9.5%) withdrew consent (one for personal reasons, one relocated country) and three (14.3%) withdrew due to adverse events (Figure 1). All patients had a documented molecular confirmation of the diagnosis of GD by *GBA1* whole gene sequencing and demonstration of bi-allelic variants. At baseline, patient mean (range) age was 43.9 (21–68) years, and mean age at diagnosis of GD1 was 27.5 years (Table 2). Baseline mean (SD) hemoglobin concentration, platelet count, and liver and spleen volumes were 13.09 (1.24) g/dL, 125.78 (50.33) × 10^9^/L, 2.77 (0.62) %BW and 1.08 (0.81) %BW, respectively. Mean (SD) duration of exposure to study treatment (*n* = 21) was 19.04 (8.51) months (median [range]: 23.0 [0.3, 25.7] months), with a mean (SD) of 41.9 (18.5) total infusions received (median [range]: 51 [2, 57] total infusions received). Nineteen (90.5%) patients received ≥ 80% of infusions. Two patients had an extended treatment period owing to COVID-19, up to a maximum total exposure of 25.7 months. Twenty (95.2%) patients received ≥ 1 concomitant medication, 15 of whom received vitamin D supplementation.

#### 3.1.2. Bone Outcomes

There was a trend for an increase in the LS BMD Z-score over time; however, the mean change from baseline to month 24 was not statistically significant, and the primary endpoint of the study was not met. Among the patients who completed this study (*n* = 16), following treatment with velaglucerase alfa, mean (SD) LS BMD Z-scores increased from −1.93 (0.88) at baseline to −1.76 (1.00) at 24 months (*n* = 16) (Table 3), representing a mean change (SD) [95% CI] of 0.17 (0.39) [−0.04 to 0.38] (*p* = 0.1077).

To evaluate the influence of sex, time from diagnosis to treatment initiation, and splenomegaly status, the change from baseline to month 24 was examined post hoc across subgroups. Among male patients (*n* = 7), the mean (SD) LS BMD Z-score at baseline was −2.17 (0.92) and was −1.87 (1.20) after 24 months of treatment, showing an increase of 0.30 (0.52). Mean (SD) LS BMD Z-scores among females (*n* = 9) at baseline and 24 months were −1.73 (0.84) and −1.67 (0.88), respectively, with a change of 0.07 (0.25). Notably, females had a higher mean (SD) age (48.9 [14.5] years) than male patients (38.3 [12.2] years). Regarding time from diagnosis to treatment initiation, patients who initiated treatment ≤10 years after diagnosis (*n* = 7) had a greater increase in the LS BMD Z-score (0.34 [0.40]; baseline −1.80 [0.61], −1.46 [0.71] at month 24) than those who first received treatment >10 years after diagnosis (*n* = 9; 0.03 [0.35]; baseline −2.02 [1.06], −1.99 [1.16] at month 24). For 2 patients with splenomegaly, the mean (SD) change from baseline in the LS BMD Z-score was 0.85 (0.21) vs. 0.07 (0.31) in 14 patients with no splenomegaly.

Bone marrow infiltration by Gaucher cells, as assessed with the BMB score, decreased significantly from baseline to 12 and 24 months with velaglucerase alfa. The mean (SD) change in the BMB score from baseline was −3.0 (1.85) at 12 months (*p* < 0.0001) and remained stable to 24 months (Table 3).

At baseline, 10 (62.5%) patients had osteopenia and 6 (37.5%) patients had osteoporosis as defined by WHO BMD classifications (normal, osteopenia and osteoporosis) based on LS T-scores. At 24 months, WHO BMD T-scores changed from osteopenia to normal for two (12.5%) patients, and from osteopenia to osteoporosis for two (12.5%) patients. No patients changed from osteoporosis to either osteopenia or normal. All seven patients in the osteoporosis group at 24 months were aged ≥50 years and six were female.

#### 3.1.3. Efficacy Outcomes (Non-BMD)

Velaglucerase alfa was found to improve hematologic and visceral disease manifestations that are characteristic of GD, indicating patient responsiveness in general to the treatment. At baseline, mean values for both hemoglobin and platelet counts were within the normal ranges. Statistically significant improvements in hemoglobin concentrations (*n* = 18) and platelet counts (*n* = 16) were observed from baseline to month 24 (*p* < 0.01; Table 3). Similarly, liver and spleen volumes were close to normal ranges at baseline; statistically significant reductions were observed from baseline to 24 months (*n* = 15; *p* = 0.0008 and *p* = 0.0114, respectively; Table 3). Statistically significant reductions in lyso-Gb1 from baseline were observed at month 24 with velaglucerase alfa in 21 patients with evaluable data (*p* < 0.0001; Table 3). Change in lyso-Gb1 concentrations were similar for males and females (−112.66 µg/mL, *p* = 0.011 [*n* = 10] and −102.83 µg/mL, *p* = 0.0022 [*n* = 11], respectively), although baseline lyso-Gb1 concentrations were higher for males than females (187.70 µg/mL vs. 122.30 µg/mL).

#### 3.1.4. Safety Evaluations

Overall, all patients experienced at least one TEAE, the majority of which (260/270 events) were mild or moderate in severity. Eleven (52.4%) patients reported a total of 43 treatment-related TEAEs, 40 of which were related to the infusion (Table 4). Three patients reported severe treatment-related TEAEs, and no patients reported serious TEAEs related to treatment. Two (9.5%) patients reported one serious TEAE each (pyelonephritis and foot fracture). Three (14.3%) patients discontinued treatment owing to a TEAE (back pain, non-cardiac chest pain, and dizziness).

The most common TEAEs were back pain (*n* = 11; 52.4%), pain in extremity (*n* = 8; 38.1%), headache and oropharyngeal pain (*n* = 6; 28.6%; Table 4). Overall, 11 patients reported TEAEs that were considered related to the infusion of the study drug. The most frequently reported infusion-related TEAEs were back pain (*n* = 5/11) and pain in extremity (*n* = 4/11). All other study drug-related TEAEs were reported by ≤2 patients.

### 3.2. Pooled Analyses

#### 3.2.1. Patient Demographics

**Figure 2:**
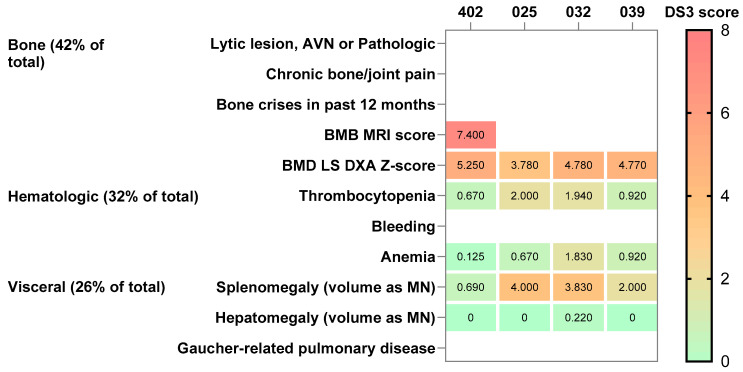
Gaucher disease severity (DS3) scores at baseline. AVN, avascular necrosis; BMB, bone marrow burden; BMD, bone mineral density; DXA, dual-energy X-ray absorptiometry; LS, lumbar spine; MN, multiple of normal; MRI, magnetic resonance imaging.

**Figure 3:**
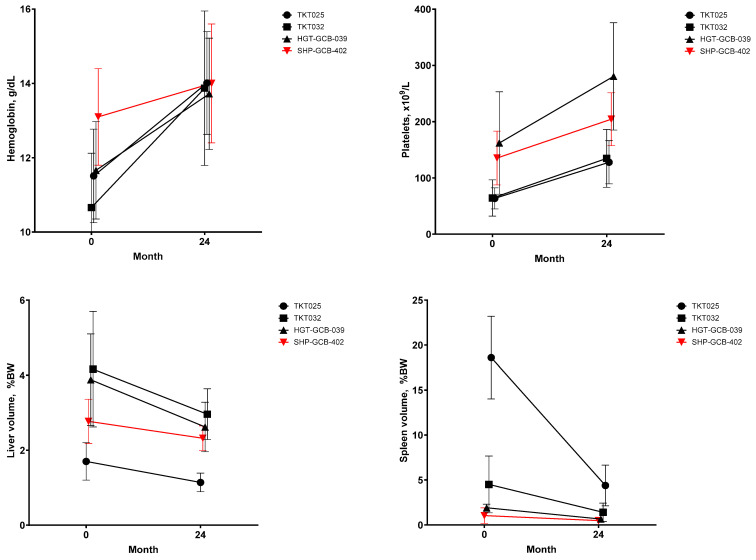
Change in hematologic and visceral parameters. BW, body weight.

Data from 40 patients treated with velaglucerase alfa (naïve at treatment initiation) across the three prior clinical trials were evaluated in pooled analyses (Table 5). Patients in the pooled prior trials were younger on average (36.8 years) than those from the SHP-GCB-402 study (43.9 years, Table 2 and Table 5). Patients in the pooled prior trials had higher baseline BMD Z-scores (−1.76 vs. −1.93, Table 3 and Table 5), and most patients in both samples had osteopenia or osteoporosis at baseline (Table 2 and Table 5). Patients in the pooled sample also had more severe GD-related hematologic and visceral symptoms at baseline than those from SHP-GCB-402 (Figure 2 and Figure 3), highlighting differences in the study population compared with patients enrolled in SHP-GCB-402 (Table S1).

#### 3.2.2. Bone Outcomes

**Figure 4:**
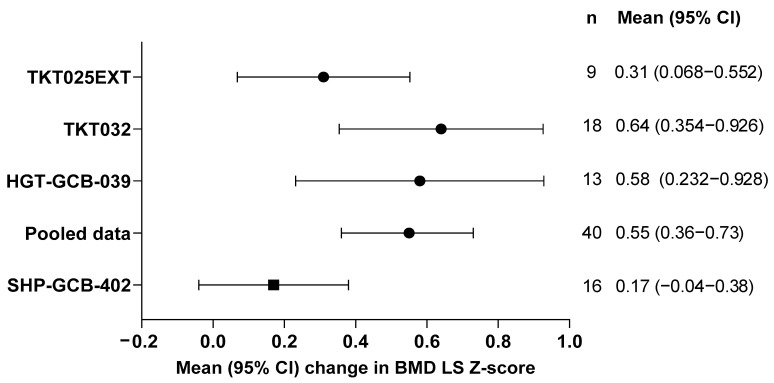
Change in BMD LS Z-score: pooled analyses. The pooled analysis includes data from TKT025EXT, TKT032/HGT-GCB-044, and HGT-GCB-039/044. BMD, bone mineral density; CI, confidence interval; LS, lumbar spine.

Similar improvements in LS BMD Z-scores were observed in patients from all three prior clinical studies. Mean (SD) change from baseline to month 24 in the LS BMD Z-score was 0.31 (0.37) in TKT025/EXT (*n* = 9; *p* = 0.0365), 0.64 (0.62) in TKT032 (*n* = 18; *p* = 0.0004), and 0.58 (0.64) in HGT-GCB-039 (*n* = 13; *p* = 0.0063). For pooled data, the mean (SD) change in the BMD LS Z-score from baseline to month 24 was 0.55 (0.58; *p* < 0.0001, *n* = 40) compared with 0.17 (0.39; *p* = 0.1077, *n* = 16) in the SHP-GCB-402 study (Figure 4). BMB was not assessed in patients ≥ 18 years of age in the TKT032, HGT-GCB-039, and HGT-GCB-044 studies.

#### 3.2.3. Hematologic and Visceral Efficacy Endpoints

Compared with the SHP-GCB-402 study, patients from the TKT025, TKT032 and HGT-GCB-039 studies had lower mean hemoglobin concentrations and higher mean spleen volumes at baseline (Table 2 and Table 5). Across all four velaglucerase alfa clinical trials, improvements in hematologic and visceral parameters were observed from baseline to month 24 (Figure 3). After 24 months of velaglucerase alfa, mean hemoglobin concentrations ranged from 13.7 to 14.0 g/L across the four studies. Greater increases were observed in studies with lower mean baseline hemoglobin concentrations compared with those with near-normal baseline concentrations. The mean spleen volume decreased to <5% (spleen volume as percentage of body weight) in all four studies after 24 months of treatment with velaglucerase alfa, with greater decreases observed for studies with higher baseline spleen volumes.

#### 3.2.4. Safety Evaluations

Safety data from the 40 patients treated with velaglucerase alfa across the three prior clinical trials showed that most TEAEs with velaglucerase alfa to be mild or moderate in severity. Similar to the SHP-GCB-402 study, most drug-related TEAEs were infusion-related. There was one treatment-related serious adverse event (in study HCT-GCB-039) and no withdrawals in TKT025, TKT032 or HCT-GCB-039. One patient in TKT032 developed antibodies. There was no effect on the clinical efficacy of velaglucerase alfa in this patient and no drug-related TEAEs were reported.

The most common TEAEs reported in >10% patients across the three prior clinical trials and in the SHP-GCB-402 study were headache, arthralgia, back pain, nasopharyngitis, pain in extremities, dizziness, myalgia, and asthenia. TEAEs occurring frequently in the three prior trials but in <10% patients in SHP-GCB-402 included influenza, rhinitis, urinary tract infection, abdominal pain, pyrexia, pharyngolaryngeal pain, injury, cough, vomiting, bone pain, and diarrhea. One patient from TKT032 who developed neutralizing antibodies only reported otitis media acute as an AE, which was considered unrelated to velaglucerase alfa treatment.

## 4. Discussion

In this phase 4 study of treatment-naïve adults with GD1, velaglucerase alfa treatment improved bone-related endpoints such as BMB score (measuring bone marrow infiltration by Gaucher cells) over 24 months. Although the change from the baseline LS BMD Z-score to 24 months was not statistically significant, a numerical mean (SD) improvement of 0.17 (0.39; *p* = 0.1077, *n* = 16) was achieved, which may have reached statistical significance with a larger sample. Consistent with results from the long-term extension study of two phase 3 clinical trials of velaglucerase alfa [14], there were significant improvements in hematologic and visceral endpoints, with no new safety concerns. The majority of TEAEs considered related to treatment in SHP-GCB-402 were infusion-related and most were mild to moderate in severity.

In contrast to results from the current study, but as demonstrated in the pooled analyses, there was a statistically significant BMD LS Z-score improvement from baseline to month 24 reported as exploratory endpoints in 31 patients with GD1 from the long-term, multicenter, pivotal trial extension study [14] maintained up to month 63, and in 9 patients with GD1 from the phase 1/2 velaglucerase alfa extension study maintained up to 69 months [15]. In addition, real-world data collected from 34 patients with GD1 from the Gaucher Outcome Survey observational study (NCT03291223) [26,27] reported improvements in BMD LS Z-scores from baseline after 2 and 8 years of velaglucerase alfa-only treatment, irrespective of age (older or younger than 50 years), sex, and baseline BMD [28] (data reported in WORLD Symposium 2025 presentation).

The reason for outcomes from the SHP-GCB-402 study being inconsistent with the pooled sample of previous velaglucerase alfa studies may lay with the differences in study populations. The SHP-GCB-402 study population consisted of patients with GD1 with low BMD, defined by an LS BMD Z-score or T-score of <−1 with no specific inclusion criteria relating to the presence or severity of hematologic or visceral manifestations of GD1, and no requirement for previous GD-specific treatment in the previous 12 months. As a result, this study included patients with mild visceral and hematologic disease severity with a lower potential for improvement, coupled with substantial BMD loss. In contrast, the populations of the TKT025, TKT032, and HGT-GCB-039 studies had a younger mean age, had more severe hematologic and visceral symptoms and less severe bone involvement at baseline (based on the disease severity scoring system for GD) than those in the SHP-GCB-402 study, further supporting the likelihood of non-GD-related bone pathology in the present study.

The post hoc analyses of changes in LS BMD Z-scores in SHP-GCB-402 highlighted specific subgroups that were driving the changes observed in the overall sample. Male patients had notably higher increases in LS BMD Z-scores at 24 months than female patients, potentially related to the fact that male patients were considerably younger than female patients (38.3 years vs. 48.9 years). Patients who had a shorter time between diagnosis and treatment also had greater improvements in LS BMD Z-scores at 24 months, supporting the notion that earlier treatment initiation can result in better outcomes [7,18]. Patients with splenomegaly had substantially greater increases in LS BMD Z-scores relative to those without. However, the majority of the sample did not have splenomegaly, indicating that the sample may have comprised patients with less severe GD [29].

A significant improvement in BMB score, a measure of bone marrow infiltration in GD, in SHP-GCB-402 is discordant with the nonsignificant improvement over time in LS BMD Z-scores. The BMB score at baseline ranged from 4 to 11, indicating some bone marrow infiltration in all patients. Further, the reduction in mean BMB score achieved with velaglucerase alfa treatment in this study (baseline of 7.8 decreasing by −3.0 to 4.8 at 24 months) was similar in magnitude to that reported by Elstein et al. (reduction of −2.9 at 24 months from a baseline of 5.8) with velaglucerase alfa in patients with GD1 [30], suggesting that BMB was related to GD pathology in this study.

BMD, as assessed by DXA, is widely considered to be a reliable measure of bone strength [9,10]. BMB, which relies on images from the LS and femora, is assessed using conventional MRI sequences and is the most commonly used scoring system in routine clinical practice due to its simple application [31]. Our study showed that there was a significant positive effect of velaglucerase alfa on BMB, but the observed effect on BMD was not significant. This result is consistent with a retrospective study of 128 patients with GD that found no correlation between BMB scores and BMD scores [32]. BMB, typically measured by MRI, reflects the degree of cellular infiltration or expansion within bone marrow such as Gaucher or malignant cells, whereas BMD, measured by DXA, reflects the mineral content and structural integrity of bone, which may not recover even if infiltration improves [7]. Therefore, it is possible that GD-specific treatments may lead to a reduction in BMB without a temporally related corresponding improvement in BMD. In SHP-GCB-402, velaglucerase alfa treatment resulted in relatively quick reductions in marrow infiltration, whereas BMD changes may occur more slowly or not at all due to chronic skeletal damage in the patient cohort. Thus, improvements in BMB may serve as an early marker of therapeutic efficacy even in the absence of changes in BMD.

Despite a relatively mild GD phenotype, with hemoglobin concentration, platelet counts, and liver and spleen volumes close to accepted normal values, treatment with velaglucerase alfa resulted in statistically significant improvements in hematologic and visceral outcomes in the SHP-GCB-402 study, although of a smaller magnitude than achieved in the three previous studies. This is explained by the presence of more severe hematologic and visceral parameters at baseline disease in the phase 1/2 and phase 3 studies. The proportion of patients reporting TEAEs, and the most frequently occurring TEAEs, were consistent with previous velaglucerase alfa studies, with small differences [12,14,20]. The occurrence of back pain, a common adverse reaction reported with velaglucerase alfa treatment in patients with GD1 [33], was more frequent in the current study compared with previous velaglucerase alfa studies, which may be explained by small population sizes, the possibility of non-GD-related bone disease at baseline, and/or progression of GD-related or GD-independent bone disease.

This study has limitations that should be considered when interpreting these results. The lack of inclusion/exclusion criteria relating to a minimum threshold for non-skeletal GD-related symptoms permitted the inclusion of patients with mild hematologic and visceral symptoms, among whom the impact of treatment might be expected to be less pronounced. In addition, the recruited sample in SHP-GCB-402 was older than those in previous clinical programs. The results might also have been impacted by factors that potentially affect bone mineralization, such as, but not limited to, tobacco use, nutrition, exercise, vitamin D supplementation, and concomitant medications [34]. The small sample size also means that results might not be generalizable to all patients with GD, and may also have contributed to the change from the baseline LS BMD Z-score at 24 months failing to reach statistical significance. Finally, ongoing challenges associated with managing skeletal manifestations in GD include the need for the standardization of assessment protocols to improve data reliability and optimize patient outcomes.

## 5. Conclusions

Findings from this study and from a pooled analysis of previously collected clinical data confirm that velaglucerase alfa continues to demonstrate efficacy in clinical outcomes with a consistent safety profile and has beneficial effects on bone disease. Our study underscores the importance of considering additional comorbidities and other confounding factors in patients with GD1 and the need for careful management of bone health in patients with GD. In cases where a considerable component of bone pathology may not be GD-related, other bone therapy such as bisphosphonate should be considered. Early treatment with velaglucerase alfa is important to mitigate GD-related bone morbidity; delayed treatment in patients with significant bone involvement and non-specific therapy may result in a suboptimal bone response. Holistic treatment of patients with GD is important to ensure that symptoms of concurrent conditions are not incorrectly ascribed to GD or its treatment.

## Figures and Tables

**Table 1:** Study details.

	TKT025/TKT025EXT	TKT032/HGT-GCB-044	HGT-GCB-039/044	SHP-GCB-402
Clinicaltrials.gov registration	NCT00391625	NCT00430625	NCT00553631	NCT02574286
Phase	1/2	3	3	4
Study period	25 April 2004–18 April 2005 3 February 2005–23 December 2011	15 February 2007–1 April 2009 13 March 2008–28 December 2012	29 January 2008–5 May 2009 13 March 2008–28 December 2012	7 June 2016–30 November 2020
Location (number of study centers per country)	Israel (1)	Argentina (1), Israel (1), Paraguay (1), Russia (1), Tunisia (1)	Argentina (1), India (3), Israel (1), Paraguay (1), Russia (1), Spain (1), Tunisia (1), UK (1), US (1)	Israel (2), Spain (1), UK (1), US (4)
Study duration	84 months total (24-month data used in this analysis)	12 months total (24-month data used in this analysis)	24 months total	24 months
Velaglucerase alfa dose	15–60 U/kg	45 or 60 U/kg EOW	60 U/kg EOW	60 U/kg EOW
Key study inclusion criteria	≥18 years Confirmed GD1 Disease-related anemia and thrombocytopenia No GD-specific treatment in previous 12 months Platelet count less than lower limit of normal GD-related anemia defined as hemoglobin levels of at least 1 g/dL below the normal limit for age and sex	≥2 years Confirmed GD1 Disease-related anemia No GD-specific treatment in previous 30 months GD-related anemia defined as hemoglobin levels of at least 1 g/dL below the normal limit for age and sex Moderate splenomegaly by palpation AND/OR GD-related thrombocytopenia (platelet count < 90 × 10^3^/mm^3^ AND/OR GD-related readily palpable enlarged liver	≥2 years Confirmed GD1 No GD-specific treatment in previous 12 months No erythropoietin in previous 6 months GD-related anemia defined as hemoglobin concentration below the local laboratory’s lower limit of normal for age and sex Moderate splenomegaly by palpation AND/OR GD related thrombocytopenia (platelet count ≥ 120 × 10^3^/mm^3^) AND/OR GD enlarged liver by palpation	≥18–≤70 years Confirmed GD1 LS BMD Z-score less than <−1 or BMD T-score of < −1 as measured by DXA No GD-specific or osteoporosis-specific treatment or erythropoietin in previous 12 months No hematologic or visceral related inclusion criteria
Key study exclusion criteria	Splenectomy	Splenectomy	Antibody-positive to imiglucerase or velaglucerase alfa at screening	Splenectomy Severe joint-associated bone damage
Key inclusion criteria for the pooled analysis	Treatment naïve at start of first study period Available baseline and 24-month LS BMD Z-score data Age ≥ 18 years at start of first study period			N/A

BMD, bone mineral density; DXA, dual-energy X-ray absorptiometry; EOW, every other week; GD1, type 1 Gaucher disease; LS, lumbar spine; N/A, not applicable.

**Table 2:** Baseline characteristics: SHP-GCB-402.

	*N* = 21
Sex, *n* (%)	
Male	10 (47.6)
Female	11 (52.4)
Age, all patients, years	
Mean (SD)	43.9 (14.2)
Median (range)	46 (21–68)
Age, males, years	
Mean (SD)	38.3 (12.2)
Median (range)	37 (21–60)
Age, females, years	
Mean (SD)	48.9 (14.5)
Median (range)	50.0 (22–68)
Age at GD1 diagnosis, years	
Mean (SD)	27.5 (15.2)
Median (range)	27.0 (3.9–59.5)
Time from diagnosis to treatment start, years	
Mean (SD)	16.3 (13.2)
Median (range)	22.1 (0.08–34.8)
*GBA1* genotype, *n* (%)	
N409S(N370S)/N409S(N370S)	11 (52.4)
N370S/V394L	1 (4.8)
N409S(N370S)/D448H/H294Q	2 (9.5)
N409S(N370S)/L29FS(84GG)	1 (4.8)
N409S(N370S)/L483P(L444P)	1 (4.8)
N409S(N370S)/UNK/L15S/S16G	1 (4.8)
N409S(N370S)/Y412*	1 (4.8)
Missing	3 (14.3)
Race, *n* (%)	
Asian	0
Black or African American	0
White	20 (95.2)
>1 race	1 (4.8)
Weight, kg	
Mean (SD)	68.58 (16.53)
Median (range)	74.1 (44.0–109.1)
BMD, Z-score	
Mean (SD)	−2.11 (0.90)
Median (range)	−2.00 (−4.2 to −0.5)
BMD, g/cm^2^	*N* = 20
Mean (SD)	0.81 (0.10)
Median (range)	0.79 (0.59–1.00)
BMB, score	*N* = 17
Mean (SD)	8.2 (2.39)
Median (range)	9 (4, 11)
Hemoglobin, g/dL	
Mean (SD)	13.09 (1.24)
Median (range)	13.10 (10.85–15.25)
Platelet count, ×10^9^/L	
Mean (SD)	125.78 (50.33)
Median (range)	127.00 (52.0–239.0)
Vitamin D 25(OH)D, nmol/L	
Mean (SD)	79.20 (31.46)
Median (range)	85.0 (10–145)
Vitamin D 1,25 dihydroxy, ng/mL	
Mean (SD)	53.18 (18.64)
Median (range)	56.50 (20.5–111.0)
Liver volume, %BW	
Mean (SD)	2.77 (0.62)
Median (range)	2.77 (1.86–4.16)
Spleen volume, %BW	
Mean (SD)	1.08 (0.81)
Median (range)	0.866 (0.22–3.21)
WHO BMD classification, *n* (%)	
Osteopenia	10 (62.5)
Osteoporosis	6 (37.5)

BMB, bone marrow burden; BMD, bone mineral density; BW, body weight; GD1, type 1 Gaucher disease; SD, standard deviation; 25(OH)D, 25-hydroxy vitamin D; WHO, World Health Organization; * indicates a wildtype allele.

**Table 3:** Mean (SD) change in efficacy endpoints and biomarkers from baseline to 24 months: SHP-GCB-402.

	*n*	Mean (SD)			*p*-Value (*t* Test)
		Baseline	24 Months	Change from Baseline [95% CI]	
LS BMD Z-score	16	−1.93 (0.88)	−1.76 (1.00)	0.17 (0.39) [−0.04 to 0.38]	0.1077
LS BMD g/cm^2^	16	0.82 (0.11)	0.83 (0.13)	0.01 (0.05) [−0.02 to 0.04]	0.3840
Total BMB score	13	7.8 (2.61)	4.8 (1.21)	−3.0 (2.27) [−4.4 to −1.6]	0.0005
Hemoglobin, g/dL	18	13.1 (1.30)	14.0 (1.60)	0.90 (1.23) [0.29 to 1.51]	0.0066
Platelet count	16	135.3 (47.9)	204.4 (47.11)	69.16 (53.45) [40.67 to 97.64]	0.0001
Liver volume, %BW	15	2.77 (0.59)	2.32 (0.33)	−0.45 (0.40) [−0.67 to −0.22]	0.0008
Spleen volume, %BW	15	1.04 (0.86)	0.49 (0.20)	−0.56 (0.74) [−0.97 to −0.15]	0.0114
Lyso-Gb1, ng/mL	21	153.44 (99.57)	45.93 (74.94)	−107.51 (77.97) [−143.01 to −72.02]	<0.0001

For change from baseline, only patients with a value at both the baseline visit and the month 24 post-baseline visit are included. BMB, bone marrow burden; BMD, bone mineral density; BW, body weight; CI, confidence interval; LS, lumbar spine; SD, standard deviation.

**Table 4:** Summary of adverse events: SHP-GCB-402 (safety set).

*N* (%)	SHP-GCB-402 (*n* = 21)
Any TEAE	21 (100)
Drug-related TEAE	11 (52.4)
Infusion-related TEAE	11 (52.4)
Severe TEAE	6 (28.6)
Drug-related severe TEAE	3 (14.3)
Serious TEAE	2 (9.5)
Drug-related serious TEAE	0
TEAE leading to discontinuation	3 (14.3)
Death	0
Most frequent TEAEs (>10% patients)	
Back pain	11 (52.4)
Pain in extremity	8 (38.1)
Headache	6 (28.6)
Oropharyngeal pain	6 (28.6)
Arthralgia	5 (23.8)
Dizziness	5 (23.8)
Fatigue	5 (23.8)
Nasopharyngitis	5 (23.8)
Myalgia	4 (19.0)
Vitamin D deficiency	4 (19.0)
Asthenia	3 (14.3)
Eye pain	3 (14.3)
Gastroesophageal reflux disease	3 (14.3)
Musculoskeletal pain	3 (14.2)
Nausea	3 (14.2)
Rhinorrhea	3 (14.2)

TEAE, treatment-emergent adverse event.

**Table 5:** Baseline patient characteristics: pooled analyses.

	TKT025/ TKT025EXT (*n* = 9)	TKT032/ HGT-GCB -044 (*n* = 18)	HGT-GCB-039/ 044 (*n* = 13)	Pooled Data (*n* = 40)
Sex, *n* (%)				
Male	5 (55.6)	10 (55.6)	5 (38.5)	20 (50.0)
Female	4 (44.4)	8 (44.4)	8 (61.5)	20 (50.0)
Mean (range) age, all patients, years	43.4 (18–69)	32.7 (19–62)	37.9 (18–60)	36.8 (18–69)
Mean (SD) age, females, years	36.0 (19.0)	29.1 (10.4)	40.0 (12.4)	34.9 (13.4)
Median (range) age, females, years	33.0 (18–60)	28 (19–52)	41.5 (19–60)	31.0 (18–60)
Mean (SD) age, males, years	49.4 (18.6)	35.5 (13.8)	34.4 (16.0)	38.7 (16.0)
Median (range) age, males, years	56.0 (25–69)	29.5 (24–62)	37 (18–58)	34.5 (18–69)
Mean (SD) weight, kg	61.5 (9.9)	62.6 (13.7)	59.9 (11.4)	61.5 (12.0)
*GBA1* genotype, *n* (%)				
L444P/Other	0	1 (5.6)	0	1 (2.5)
N370S/84GG	0	0	1 (7.7)	1 (2.5)
N370S/L444P	1 (11.1)	1 (5.6)	1 (7.7)	3 (7.5)
N370S/N370S	4 (44.4)	7 (38.9)	5 (38.5)	16 (40.0)
N370S/Other	4 (44.4)	7 (38.9)	4 (30.8)	15 (37.5)
Other/Other	0	2 (11.1)	2 (15.4)	4 (10.0)
Mean (SD) hemoglobin concentration	11.87 (1.18)	10.66 (1.46)	11.66 (1.31)	11.26 (1.43)
Mean (SD) platelet count	63.39 (18.80)	65.12 (33.03)	162.08 (91.05)	97.04 (72.96)
Mean (SD) liver volume, %BW	3.94 (1.28)	4.16 (1.54)	3.88 (1.22)	4.02 (1.36)
Mean (SD) spleen volume, %BW	3.59 (0.99) [*n* = 9]	4.51 (3.16) [*n* = 18]	1.91 (0.40) [*n* = 5]	3.85 (2.58) [*n* = 32]
Mean (SD) BMD LS Z-score	−1.49 (1.1)	−1.95 (1.1)	−1.65 (1.1)	−1.76 (1.1)
WHO classification, *n* (%)				
Normal	1 (11.1)	3 (16.7)	2 (15.4)	6 (15.0)
Osteopenia	7 (77.8)	9 (50.0)	6 (46.2)	22 (55.0)
Osteoporosis	1 (11.1)	6 (33.3)	5 (38.4)	12 (30.0)

BMD, bone mineral density; BW, body weight; LS, lumbar spine; SD, standard deviation; WHO, World Health Organization.

## Data Availability

The datasets, including the redacted study protocol, redacted statistical analysis plan, and individual participants data supporting the results of the completed study, will be made available after the publication of final study results within 3 months from initial request to researchers who provide a methodologically sound proposal. The data will be provided after its de-identification, in compliance with applicable privacy laws, data protection, and requirements for consent and anonymization.

## Supplementary Materials

The following supporting information can be downloaded at: https://www.mdpi.com/article/10.3390/jcm15072537/s1, Table S1: Baseline comparisons between patients in SHP-GCB-402 and in the pooled clinical studies.

## Abbreviations

The following abbreviations are used in this manuscript:

AVN	Avascular necrosis
BMB	Bone marrow burden
BMD	Bone mineral density
BW	Body weight
CI	Confidence interval
DS3	Disease severity score
DXA	Dual-energy X-ray absorptiometry
EOW	Every other week
*GBA1*	Glucocerebrosidase gene
GD	Gaucher disease
GD1	Type 1 Gaucher disease
LS	Lumbar spine
MN	Multiple of normal
MRI	Magnetic resonance imaging
SD	Standard deviation
TEAE	Treatment-emergent adverse event
WHO	World Health Organization
