# Supplementary material for: Improvement of Bone Mineral Density in Patients with Type 1 Gaucher Disease Treated with Velaglucerase Alfa: Results from Clinical Studies
Source: J Clin Med. 2026 Mar 26;15(7):2537. doi: 10.3390/jcm15072537 (PMC13073715; doi:10.3390/jcm15072537)
Supplement: Supplementary file 1 [file jcm-15-02537-s001.zip › jcm-4133690-supplementary.pdf]

Supplementary Material

Table S1. Baseline comparisons between patients in SHP-GCB-402 and in the pooled clinical studies.

	**Pooled (*N* = 40)**	**SHP-GCB402 (*N* = 16)**	***p*-Value**
Age, mean (SD), years	36.78 (14.71)	45.31 (15.07)	0.0565
Female, *n* (%)	20 (50.0)	9 (56.3)	0.6724
Age of females, mean (SD), years	34.85 (13.38)	48.91 (14.52)	0.0230
Weight, mean (SD), kg	61.48 (11.95)	67.80 (17.65)	0.1264
Z-score, mean (SD)	-1.75 (1.02)	-1.93 (0.88)	0.5560
WHO category, *n* (%)			
Normal	6 (15.0)	1 (6.3)	
Osteopenia	22 (55.0)	10 (62.5)	0.8351
Osteoporosis	12 (30.0)	5 (31.3)	
Hemoglobin, mean (SD)	11.27 (1.44)	13.11 (1.14)	<0.0001
Platelets, mean (SD)	95.80 (72.43)	137.43 (49.45)	0.0455
Liver volume, mean (SD), %BW	4.02 (1.36)	2.72 (0.59)	0.0006
Spleen volume, mean (SD), %BW	3.85 (2.58)	1.01 (0.85)	<0.0001

BW, body weight; SD, standard deviation; WHO, World Health Organization.
